# Creating a multi‐centre Amyloid PET dataset for DLB patients: Design and Methodology

**DOI:** 10.1002/alz70862_109944

**Published:** 2025-12-23

**Authors:** Ariane Bollack, Beatrice Orso, Mahnaz Shekari, Cecilia Boccalini, Valentina Garibotto, Giovanni B Frisoni, Lisa Quenon, Bernard J Hanseeuw, Val J Lowe, Lyduine E. Collij, Christopher Buckley, Alan J Thomas, John T O'Brien, Gill Farrar

**Affiliations:** ^1^ UCL Centre for Medical Image Computing, London UK; ^2^ GE HealthCare, Chalfont St Giles, Buckinghamshire UK; ^3^ University of Genoa, Genoa, Genoa Italy; ^4^ Barcelonaβeta Brain Research Center (BBRC), Pasqual Maragall Foundation, Barcelona Spain; ^5^ University of Geneva, Geneva, Switzerland Switzerland; ^6^ Faculty of Medicine, University of Geneva, Geneva, Geneva Switzerland; ^7^ Geneva Memory Center, Geneva University Hospitals and University of Geneva, Geneva Switzerland; ^8^ Institute of Neuroscience, UCLouvain, Brussels Belgium; ^9^ Gordon Center for Medical Imaging, Massachusetts General Hospital, Harvard Medical School, Boston, MA USA; ^10^ Institute of Neuroscience, Université Catholique de Louvain, Brussels Belgium; ^11^ Mayo Clinic, Rochester, MN USA; ^12^ Amsterdam UMC location VUmc, Amsterdam, Amsterdam Netherlands; ^13^ Clinical Memory Research Unit, Department of Clinical Sciences Malmö, Faculty of Medicine, Lund University, Lund Sweden; ^14^ Newcastle University, Newcastle upon Tyne, Newcastle UK; ^15^ University of Cambridge, Cambridge, Cambridgeshire UK

## Abstract

**Background:**

Dementia with Lewy Bodies (DLB) is often misdiagnosed or conflated with Alzheimer’s Disease (AD) due to overlapping clinical presentations and neuropathological features. The presence of AD‐like features correlates with accelerated cognitive decline and a worse overall prognosis. Compared to AD, studies suggest that amyloid uptake in DLB may spare the occipital lobe. This project aims to investigate whether amyloid PET could be leveraged to differentiate between AD and DLB patients, with the goal of improving diagnostic accuracy and supporting patient stratification and safety in anti‐amyloid trials.

**Method:**

This study involves collaboration across six centres (University of Geneva, BioFINDER, AMPLE, AMYPAD PNHS, MCSA, GEHC), each contributing amyloid PET imaging data from patients diagnosed with DLB based on clinical or neuropathological assessments. Moreover, each center provided an additional subset of AD and healthy controls, then matched to DLB samples based on age/sex/APOE genotype/education for comparison. A sub analysis will be performed on patients with evidence of both DLB and AD markers. Given the relatively small sample size, key aspect of the methodological approach is to reduce technical sources of variability. The processing workflow includes rigorous quality control, formatting, normalisation to MNI space, harmonisation (differential smoothing), and MRI‐based segmentation.

**Result:**

The dataset currently includes amyloid PET scans (acquired using [^11^C]PiB, [^18^F]Flutemetamol or [^18^F]Florbetapir) and MRI scans from ∼130 DLB patients, along with ∼150 AD patients and ∼150 controls. In addition, 99 patients positive for the CSF α‐Synuclein Seeding Amplification Assay were included. Patient data were anonymized, and a harmonised database was created, including demographic characteristics, clinical history, and results from neuropsychological assessments.

**Conclusion:**

The creation of a standardized, multi‐centre amyloid PET dataset for DLB will enable the study of amyloid PET patterns at the global, regional and voxel level, in comparison with uptake in AD patients and controls. This dataset will serve as a valuable resource for investigating the neuropathological underpinnings of DLB, improving diagnostic accuracy, and potentially guiding patient stratification in anti‐amyloid trials. Efforts to integrate [^18^F]Florbetaben and additional data are ongoing.

